# *Legionella* and Biofilms—Integrated Surveillance to Bridge Science and Real-Field Demands

**DOI:** 10.3390/microorganisms9061212

**Published:** 2021-06-03

**Authors:** Ana Pereira, Ana Rosa Silva, Luis F. Melo

**Affiliations:** LEPABE—Laboratory for Process Engineering, Environment, Biotechnology and Energy, Faculty of Engineering, University of Porto, Rua Dr. Roberto Frias, 4200-465 Porto, Portugal; arms@fe.up.pt (A.R.S.); lmelo@fe.up.pt (L.F.M.)

**Keywords:** *Legionella*, *Legionella* prevention, biofilms, field-based studies, biofilm monitoring, engineered water systems, integrated management

## Abstract

*Legionella* is responsible for the life-threatening pneumonia commonly known as Legionnaires’ disease or legionellosis. Legionellosis is known to be preventable if proper measures are put into practice. Despite the efforts to improve preventive approaches, *Legionella* control remains one of the most challenging issues in the water treatment industry. Legionellosis incidence is on the rise and is expected to keep increasing as global challenges become a reality. This puts great emphasis on prevention, which must be grounded in strengthened *Legionella* management practices. Herein, an overview of field-based studies (the system as a test rig) is provided to unravel the common roots of research and the main contributions to *Legionella’s* understanding. The perpetuation of a water-focused monitoring approach and the importance of protozoa and biofilms will then be discussed as bottom-line questions for reliable *Legionella* real-field surveillance. Finally, an integrated monitoring model is proposed to study and control *Legionella* in water systems by combining discrete and continuous information about water and biofilm. Although the successful implementation of such a model requires a broader discussion across the scientific community and practitioners, this might be a starting point to build more consistent *Legionella* management strategies that can effectively mitigate legionellosis risks by reinforcing a pro-active *Legionella* prevention philosophy.

## 1. Introduction

Legionnaires’ disease (LD), also called legionellosis, is a worldwide public health concern caused by the waterborne pathogen, *Legionella* [1,2]. Legionellosis is a severe form of pneumonia with a fatality rate of approximately 10% [3,4]. In Europe and in the United States (US), it is known to be responsible for the death of around 15–20 persons per 10 million inhabitants, annually [5,6,7]. LD infections occur mostly via inhalation of small droplets of water (aerosols) contaminated with virulent bacteria strains [8]. *Legionella* is naturally present in fresh waters, yet it is in engineered water systems (e.g., cooling tower, premise plumbing, etc.) that it finds the ideal conditions to proliferate to concentrations that can endanger people’s lives [8].

The number of legionellosis reported cases in 2017 shows an incidence rate of 1.8 and 2.2 per 100,000 inhabitants in Europe [5] and in the US [6], respectively. In the United States, *Legionella* is already responsible for the highest number of deaths among waterborne pathogens [9]. LD, besides being a significant societal problem, also represents a high economic cost to the health care system [10]. However, legionellosis incidence and its associated health risks are known to be increasing [11,12,13], due to global challenges such as urbanization, ageing populations, climatic changes, or circular economy approaches [12,13]. The number of people diagnosed with legionellosis will rise to around 2.5 billion by 2050 in urbanized centers [14,15], and the need for more climatization solutions will also grow [11]. Circular economy and water reuse, while necessary, will likely increase the number of water systems and their complexity and will change water consumption patterns [16]. As foreseen by Walker et al. [17], climate change is also expected to favor the rise of waterborne diseases. Climate change is not only restricted to the temperature increase of the planet but is also related to seasonality pattern shifts or more frequent extreme weather events. For example, higher precipitation is known to potentiate the risk of sporadic Legionnaires’ disease [18,19].

Legionellosis is considered a preventable illness [20] if proper *Legionella* control measures [21,22] are put into practice at water systems. Prevention is actually the great emphasis of most worldwide guidance and legislation [23,24,25] as an underlying principle of Water Safety Plans (WSPs). WSPs must be advisedly established to cover different aspects of *Legionella* control as well as the uniqueness and specificities of the water system. A key component of these plans is routine monitoring aiming, among others, to access the efficacy of a water management program and to identify malfunctioning of the system [23,25,26]. However, too often these monitoring strategies are over-dependent on *Legionella* discrete water sampling outputs [10,27,28]. As will be discussed, this becomes a serious bottleneck for *Legionella* prevention.

Across the scientific literature, there are several works dedicated to the study of *Legionella* behavior directly within real-field water systems, which will be the focus of this review. Digging into these field-based studies (as they will be called in this review) allows us to identify some common areas of research: *Legionella* widespread in water systems, microbial control strategies, and the role of the microbiome and bacterial communities in *Legionella* proliferation. Unsurprisingly, most of these field-based studies are grounded in *Legionella* screenings in the water, while exploring the potential of new inputs from molecular tools [29]. However, they often ignore the role of ecological niches, such as protozoa and biofilms, as critical spots for *Legionella* settlement, adaptation, and infectivity [30,31]. Biofilm sampling is actually within the scope of routine *Legionella* environmental surveillance by many reference documents [23,24,25], yet biofilm sampling and analysis lack standard sampling and analytical practices [32,33] that can give consistent and representative outputs. Online biofilm monitoring methods can provide an important contribution to the assessment of information about these attached layers, overcoming current water treatment limitations [34]. It is expected that their implementation will also have an inherently positive impact on *Legionella* control. However, there is a huge gap between biofilm monitoring’s potential added value and the adoption of these methods in scientific studies or as part of real-field practices.

While global challenges are becoming a reality, there is an urgent need to avoid their collateral effects on people’s health and to invert the increasing legionellosis incidence trend. New (or renewed) approaches for real-field *Legionella* management will depend on consistent scientific practices and findings and on disrupting ‘the same-as-usual’ approach. As such, the last section of this review will propose some pathways to bridge microbiological water screening and biofilm monitoring engineering. The definition of an integrated platform, combining discrete surface and water sampling with online, real-time monitoring, might broaden the understanding of *Legionella*’s proliferation, as well as deliver early warning information that can trigger specific calls for action. Bridging areas of knowledge is essential to fulfill the tremendous *Legionella* control challenge. Ultimately, this paper aims to contribute to the discussion of how to build more consistent and implementable preventive practices while strengthening scientific approaches.

## 2. Common Roots of Research from *Legionella* Field-Based Studies

Although the study of *Legionella* is still very recent (the disease raised up around the last quarter of the 20th century) [22], some aspects seem to consistently characterize and be relevant for *Legionella* prevalence in water systems and for further infection in human beings [35]. Legionellosis has been linked to different water systems, such as cooling towers (CT) [36,37], hospital [38,39] and hotel [40,41], water supply lines, or whirlpool spas [42,43]. All these systems have some common characteristics that favor *Legionella* settlement and growth. Apart from the complex and often unknown architectures of interconnected pipelines, equipment, pumps, and other hydraulic settings [35] that can potentially disseminate the bacteria by aerosolization, they also have very diverse conditions of the system. Warm temperatures, hydrodynamic patterns (specially stagnation) [35], water quality (pH and hardness) [44,45,46,47], corrosion and scaling (as nutrient sources) [48], and surface materials [49,50] have been linked to *Legionella* prevalence. Additionally, biocidal programs that are not properly set or managed may promote *Legionella* colonization in several installations [51,52,53,54]. Mimicking the diversity and dynamic conditions of engineered water systems is an unfeasible task in the laboratory. As such, real-field systems arise as close-to-ideal test rigs to study *Legionella* prevalence and behavior.

Therefore, in this review, the focus will be on field-based studies with the following common features: (i) they address a particular water setting; (ii) sampling and monitoring is carried out on-site; (iii) they have long timeframes (from several months to several years); (iv) they evaluate *Legionella* behavior, over time, against one or more specific conditions. Based on these criteria, Table 1 provides an overview of recent *Legionella* field-based studies, organized according to the main topics and findings of the research.

### 2.1. Main Research Topics

Three main research areas appear among field-based studies that tend to be centered on the following keywords: ‘*Legionella* widespread’, ’Microbial control strategies’, and ‘Microbiome and bacterial communities’.

These are all critical aspects related to *Legionella* persistence in man-made systems that contribute to a higher or lower legionellosis risk. The studies considered in Table 1 cover different water settings, including cooling towers [55,56,57], premise plumbing from hospitals [51,52,54,58,59,60,61,62,63,64] and hotels [45,48,64,65,66,67,68,69], residential potable water systems [46,70,71], and other facilities [72,73,74,75,76,77,78].

**Table 1:** Overview of recent *Legionella* field-based studies and their main findings.

Target of Research	Main Findings	References
***Legionella*** **Widespread**	*High Legionella prevalence regardless of the water system*	[45,46,56,57,63,67,68,69]
	*Legionella incidence seems to be seasonal dependent*	[64,69,71,73]
**Microbial Control Strategies**	*Chemical Disinfection Programs shape bacterial communities and reduce Legionella positivities (chlorination, chloramine, chlorine dioxide, copper-silver ionization, hydrogen peroxide and silver salts, NEOW)*	[51,52,54,56,61,62,66,72,73,79,80,81]
	*Effective temperature control reduces Legionella incidence*	[60,70,82]
	*Water stagnation vs. water flushing*	[48,59,65,71,78,83]
**Microbiome** **and Bacterial** **Communities**	*Bacterial communities—antagonists or promoters of Legionella persistence*	[51,55,56,58,74,76]
	*Protozoa and biofilm niches for Legionella growth*	[60,70,72,74,75,76,77]

#### 2.1.1. *Legionella* Widespread

The number of LD outbreaks has been on the rise [50], which demonstrates that *Legionella* ubiquity is a reality in man-made water systems. All studies confirm the general idea that *Legionella* spp. are widely present across engineered water networks [48,71,83] and that there is a high recovery of *L. pneumophila* [63,68] amongst *Legionella* species.

##### High *Legionella* Prevalence Regardless of the Water System

Kyritsi et al. [45] sampled 51 hotels in Greece (556 water samples taken from showers, swimming pools, taps, cooling towers, a fountain, coolers, boilers, cold-water tanks, and hot tubs) to investigate their colonization by *Legionella*. It was found that 74.5% of the hotels were colonized by *Legionella* spp., and 28% of the collected samples were positive for *Legionella* spp. Recently, Yakunin et al. [67] also found that, in 6 districts in Israel, 60% of the 169 hotels and resorts assessed in their work were positive for *Legionella* spp., and 99% of the 162 serotyped isolates belonged to *L. pneumophila*. Moreover, a high presence of *Legionella* in cooling towers (CTs) was also observed across different US regions, where Llewellyn et al. [57] reported that 40% of the sampled CTs (79 from 196) were *Legionella* spp. culture-positive and that *L. pneumophila* was the most recovered among the several strains. Other studies targeting different facilities also found *L. pneumophila* in most of the collected water samples [46,63,68].

##### *Legionella* Incidence Seems to Be Seasonal Dependent

Numerous studies have also focused on the investigation of the seasonal occurrence of *Legionella*. Ley et al. [71], Liu et al. [73], and De Giglio et al. [69] conducted long-term studies to evaluate shifts in *Legionella* counts according to the season in a residential building, tap-water premise plumbing, and in touristic recreational facilities, respectively. Although different facilities were assessed, they all found that *Legionella*-positive water samples were significantly higher in summer, compared to winter. These findings agree with a previous study conducted by Bentham et al. [84], who found that the colonization rate of 31 cooling towers in South Australia decreased to less than a half between summer and winter samplings. The authors consensually pointed out the effect of temperature as a primary reason behind their findings. Furthermore, Liu et al. [73] also linked the chlorine decay during summer to higher *Legionella* counts.

#### 2.1.2. Microbial Control Strategies

An important part of a successful *Legionella* management program will depend on the efficacy of microbiological control strategies [25]. As such, several works have investigated the effect of different control measures (conventional and new) on *Legionella* prevalence in real-field systems. Conventional approaches include disinfectant dose, temperature control, or water flushing [85]. Under this topic, most of the published studies are focused on hot water networks, probably because there is only a restricted number of control measures available, and these should fulfill the requirements of drinking water regulations (which limit, for example, the type of biocides to use), as well as comply with safety measures for users (e.g., avoid scalding).

##### Chemical Disinfection Programs Shape Bacterial Communities and Reduce *Legionella* Positivities

As demonstrated by many studies, implementing an appropriate chemical disinfection program on a routine basis can be an effective way to control microbial proliferation in water systems and mitigate LD risks [51]. For example, Paranjape et al. [56] and Mouchtouri et al. [86] found out that the effect of chlorination in cooling towers in Canada and Greece were crucial to minimizing the colonization and recolonization of *Legionella* spp. Furthermore, the continuous dosage of chlorine in CTs seems to be directly linked to a sort of microbial species selection that favors (or not) *Legionella pneumophila* presence [56]. Le Chevallier [81], when sampling 10 utilities (669 samples) comprising plant effluents and distribution systems, reinforced the importance of maintaining a chlorine residual in order to keep low *Legionella* levels, especially when the water temperature is above 18 °C.

Lytle et al. [52] evaluated a hospital where hot water flushing was used to reduce *Legionella* levels. The reemergence of bacteria led to the implementation of a monochloramine disinfection system, which was a successful technical change since a significant decrease (from 68% to 6%) in culturable *Legionella* was reported after the addition of the monochloramine to the hot water network. Similar conclusions were observed in other field-based studies with monochloramine dosage [54,62].

Vincenti et al. [61] conducted a long-term study in which *Legionella* prevalence in hospital water networks treated with chlorine dioxide was evaluated, with a positive impact on *Legionella* control. *Legionella* spp. were monitored over 4 years after the ClO_2_ implementation and were not detected in ~81% of the sampling points.

The work of Cloutman-Green et al. [80] shows that it is possible to control *L. pneumophila* incidence in a new hot water network at low temperatures (average temperature ~42 °C) using copper-silver ionization and taking advantage of the combination of the antimicrobial effect of the Cu^2+^ and Ag^+^ ions. Also, the synergistic effect of hydrogen peroxide and silver salts has been successfully applied to reduce *Legionella* incidence in a hospital hot water network [79]. The authors found a reduction in *Legionella*-positive sites from 60% to 36%, compared with previous treatment with ClO_2_. Although hydrogen peroxide has a relevant role in water treatment, its synergistic effect with silver salts enhances disinfection efficacy [87].

Neutral electrolyzed oxidizing water (NEOW) is generated through the electrolysis of sodium chloride solutions [66]. The process requires an electrochemical cell [88] where the anode and the cathode are separated by a membrane [66]. This process generates chlorine oxidants with biocidal potential [88]. NEOW, under laboratory experiments, showed to be very promising to reduce the microbial load in fresh-cut vegetables [89], as well as to show great potential in decreasing pathogen concentrations in the bulk water [90,91]. Bonetta et al. [66] investigated a hot water distribution system from a hotel where *L. pneumophila* was detected when the water was only treated with a UV lamp before distribution. To reduce *Legionella* contamination, a NEOW system was installed and samples demonstrating lower levels of *Legionella* were subsequently collected.

##### Effective Temperature Control Reduces *Legionella* Incidence

Temperature between 25 °C and 50 °C [44,50] and water stagnation are known to be critical factors for *Legionella* growth [82] and are parameters that must be carefully addressed in routine base procedures, especially those associated with building water networks. Quero et al. [60] conducted a study over 2 years in a hospital where copper-silver ionization and heat-and-flush control treatments were applied as disinfection treatments. As their effectiveness was not satisfactory, a different strategy was embraced, and the temperature was raised to 55 °C in the return pipes. Afterwards, the temperature showed to be much more stable in all areas (even on the distal points), and a decrease in *Legionella* incidence was observed, although amoebae presence had not been affected. Additionally, Gavaldà et al. [82] found in an 8-year study that the temperature of the hot water in a large hospital was a critical factor. The authors observed that a minimum of 55°C can substantially decrease *Legionella* detection. However, temperature efficacy on *Legionella* control showed to be highly dependent on the hydraulics and operation practices at the systems (e.g., suitable recirculation loops and periodic tap flushing).

Thermal disinfection is frequently coupled with other disinfection treatments, such as hyperchlorination [12] since its effectiveness alone is not enough [49] to significantly reduce *Legionella* amounts. Additionally, rapid recolonization of the system is observed after heat shock treatment [92]. This might be explained by the complex system configurations that often do not allow to maintain a constant and desired temperature in all parts of the system, nor its proper monitoring [82].

##### Water Stagnation vs. Water Flushing

Water stagnation is known to be a critical factor for water-quality decrease, with an inherent impact on *Legionella* prevalence [93]. For example, De Giglio et al. [59] assessed the microbiological water quality in an Italian hospital over 3 months during the COVID-19 lockdown. The authors found higher concentrations of *L. pneumophila* after the lockdown, which was attributed to building inoccupation. The work of Rhoads et al. [93] provides an interesting overview of the complex topic of water stagnation related to *Legionella* management. One of the strategies to minimize stagnation problems is water flushing. Water flushing—increasing water flow for a defined period of time [48]—continues to be implemented and promotes a decrease in *Legionella* levels [83]. For example, Totaro et al. [48] evaluated *Legionella* persistence on a chemically treated water network from a hospital after the installation of time flow taps (to implement programmed water flushing at the system’s dead-end branches). Before implementing the flushing procedure, *L. pneumophila* was detected in all sampling points, but after regular flushing, the levels started to decrease. Similarly to what happens with temperature, water flushing in drinking-water-related systems is usually co-adjuvanted by other strategies, such as temperature increase or chemical dosage [65,78]. Routine water flushing is particularly important when disinfectant agents are added to water systems since it assures that disinfectant levels are maintained throughout the whole system [94].

#### 2.1.3. Microbiome and Bacterial Communities

The commensal microflora present in engineered water systems strongly impacts *Legionella* survival and proliferation [30,51,56,95]. Protozoa and biofilms are naturally part of this ecosystem. A detailed discussion about their relevant role as environmental niches for *Legionella* proliferation can be found in Section 3, while the analysis herein is strictly related to the findings gathered in the field-based studies.

##### Bacterial Communities—Antagonists or Promoters of *Legionella* Persistence

The role of bacterial communities as antagonists or promoters of *Legionella* persistence in water systems has been suggested in several earlier laboratory studies [96,97,98]. In field-based systems, microbial consortia selection is intrinsically affected by the water source, as well as by operating conditions and control procedures. Paranjape et al. [56], when characterizing the bacterial communities of 18 CTs in different regions of Quebec, concluded that the water source highly affects the bacterial compositions of each CT and that the presence or absence of certain species affects *Legionella’s* detected concentration. *Pseudomonas* presence was found to have a strong negative effect on *Legionella* population, inhibiting its growth. This conclusion agrees with the former study of Llewellyn et al. [57], which also found that cooling towers with PCR-negative *Legionella* showed a significantly higher abundance of *Pseudomonadaceae*. In addition, Toze et al. [99] carried out an investigation on a drinking water facility and found that *Pseudomonas* spp. did not support *Legionella* growth. Very interestingly, Stewart et al. [98], in a laboratorial bioreactor, concluded that *L. pneumophila* could not persist in a *Pseudomonas aeruginosa* (known to be able to kill amoebae) biofilm but showed increased growth in a *Pseudomonas fluorescens* biofilm, reinforcing the idea that *Legionella* presence is highly dependent on the specific microbial communities at the ecological system.

Moreover, Tang et al. [58], Garner et al. [76], and Li et al. [74] found positive correlations between the presence of *Legionella* spp. and *Mycobacterium* spp., even though their studies were performed in different field-based settings. These findings highlight an important ecological relationship between some of the most concerning opportunistic pathogens in drinking water systems.

##### Protozoa and Biofilm Niches for *Legionella* Growth

Several authors found a relationship between *Legionella* persistence and protozoan species, such as *Acanthamoeba*, *Hartmannella*, and *Tetrahymena* [74,100,101]. For example, Gomes et al. [75] studied four drinking water treatment plants and detected in several samples significant amounts of *Legionella* spp. in the plants, especially after co-culture with *Acanthamoeba*. Higher concentrations of free *Legionella* were found in raw water than in finished water. On the other hand, *Legionella*-infected free-living amoebae concentrations increased in the water treatment plant (i.e., higher concentrations were found in the finished water than the raw water). No free *Legionella* was observed in the biofilm samples, yet they were found to be related to a higher diversity of free-living amoebae.

Complementary to water screening, some studies also collected and analyzed biofilm samples from systems surfaces, using sterile swabs. De Filippis et al. [70], Waak et al. [72], and Garner et al. [76] performed research studies in drinking water facilities and sampled water and biofilm. De Filippis et al. [70] identified *Legionella* more frequently in water rather than in biofilm samples when sampling showerheads from retirement and group homes. Indeed, while *Legionella* was positive both in water and biofilms in 21 out of 124 sampling points, *Legionella* was exclusively detected in biofilms in only 1 sampling point. Although there is no clear explanation for these findings, which are similar to the ones from Gomes et al. [75], they might be related to the biofilm-swabbing sampling and analytical protocol (as will be discussed in later sections), as well as to the specific microbial consortia found in the biofilm, which can be unfavorable to *Legionella* growth.

Very pertinently, Garner et al. [76] studied reclaimed and potable water distribution systems regarding the occurrence of five opportunistic pathogens, including *Legionella* spp. The authors concluded that microbial communities of both water systems were most abundant in the biofilms in comparison to the bulk water. Furthermore, *Legionella* spp. amounts were higher in biofilms from reclaimed systems than from potable ones. Waak et al. [72] collected water and biofilm samples from two drinking water systems, with and without residual disinfection (chloramine), from the United States and Norway, respectively. *Legionella* spp. were not detected in water and biofilm samples collected from the system with residual disinfection agent, while biofilms samples collected from the other DW system (no chloramine residual) were positive for *Legionella* spp. The conclusions of this work reinforce the idea that chloramine might react with extracellular polymeric substances [102] and could thus be more effective against bacteria since it can penetrate the microbial layers.

### 2.2. Field-Based Studies’ Added-Value and Opportunities

There is consensus in the literature that *Legionella* persistence in water systems is the result of several interplay factors associated with each system, such as, for example, water source, system design, or control measures. As such, studying *Legionella* in association with the microbial ecosystem directly in real water settings, for long periods of time, is a strongly recommended way to investigate *Legionella* as the direct output of all operational constraints of the system.

The studies overviewed in Section 2 show the common roots of the research. It is important to emphasize that the systematization shown in Table 1 only focuses on real-field system studies. This remark is particularly important regarding the sub-topic ‘Microbial Control Strategies’, where great relevancy has been given to some techniques, while others that are less reported in field-studies have not been revised. While they do not provide disruptive conclusions to the general knowledge, these findings highlight some important aspects of *Legionella* prevention in water systems. First, it might be dangerous (and is not recommended at all [24]) to address *Legionella* management under the presumption of a ‘*Legionella*-free’ system, even if routine water samplings do not detect legionellae. As discussed, *Legionella* settlement and growth is not only the result of the control measures but is also affected by the microbial communities cohabiting in the same water. Such a widespread of *Legionella* spp. among water systems, regardless of the systems’ type, specificities, or their geographical area, also means that positive *Legionella* results do not necessarily imply legionellosis cases. As such, this emphasizes the need to build adaptable and integrated preventive measures, which allow the routine surveillance of *Legionella* in water systems and mitigate legionellosis risk.

Another important reflection concerns the lack of combined *Legionella* screening in the water and at the surface. The research methodologies used in most of the field studies previously discussed are based on periodic water sampling followed by its physical, chemical, and microbiological evaluation. *Legionella* screening and quantification are usually accomplished by a culturing technique and qPCR (quantitative polymerase chain reaction) in parallel. On the other hand, only a few studies addressed biofilm (through surface swabbing or scrapping) and water analysis at the same time. In fact, many studies listed in Table 1 only refer to biofilms two or three times throughout their papers. Ignoring the role of biofilms in sheltering bacteria might raise important bias and interpretation problems, as discussed in detail in the next sections.

## 3. Key Topics That Need to Be Tackled for Effective *Legionella* Real-Field Prevention

Several aspects make *Legionella* management and prevention in water systems a very challenging task. Two of those aspects deserve particular attention.

The first aspect is linked to the ecology of the bacteria. *Legionella* is a bacteria that, despite its fastidious nutritional requirements, survives and adapts to different conditions [22,31]. The parasitic lifestyle with protozoa [100] and the synergies established in biofilms [103,104], as well as their ability to enter the viable-but-non-culturable cell (VBNC) state [105], seem to be key to *Legionella’s* successful persistence under harsh external stresses. The water systems’ complexity and extension promote the existence of different preferential spots for *Legionella* settlement and growth, which are often difficult to identify, access, and inspect [23].

The second important aspect is related to *Legionella* monitoring and control practices, which are over-reliant on single water-sampling snapshots in time that provide unrealistic pictures of the amount of *Legionella* in the water system [27].

In spite of these limitations, water-focused practices are still perpetuated in scientific studies, as discussed in the previous section. As such, an integrated reflection on these bottom-line questions will help in identifying pathways that can overcome some of the *Legionella* control bottlenecks and reinforce risk mitigation strategies.

### 3.1. Legionella a Case of Resilience

The generally accepted mechanisms/hypotheses by which legionellae is able to replicate in water systems are as follows [20,106]: (a) bulk water offers a set of conditions that favor *Legionella* replication up to high planktonic concentrations; (b) *Legionella* spp. infect free-living protozoa, such as amoebae, and multiply intracellularly within these hosts; (c) *Legionella* is sheltered in biofilms that offer protection and provide the necessary conditions for its proliferation.

As suggested by hypothesis (a), *L. pneumophila* can survive as a free-living organism, yet its ability to grow to significant concentrations without a host seems to be very limited [25]. Growing *L. pneumophila* in a laboratory is a difficult task, involving an unusual set of nutrient requirements [31,107] that are not commonly found in fresh water [22]. This seems to contradict the wide spread of the bacteria and their ability to proliferate in such oligotrophic (nutrient-scarce) environments. This apparent contradiction raises the idea that *Legionella* fulfils its nutritional needs through a parasitic-based lifestyle [25,31], more consistent with mechanisms (b) and (c). This hypothesis is further strengthened when considering the dehydration phenomena that occur when the small droplets that carry the bacteria are dispersed in the air. Given the negative effect that dehydration has on *Legionella* viability [106], it is unlikely that free *Legionella* keeps its viability and infectivity upon aerosol dispersion (a critical step for human contamination). Mechanisms (b) and (c) will be addressed in item 3.2.

Another important aspect of *Legionella* resilience is its ability to enter the VBNC state as a response to stress conditions such as high temperature [53], biocides [108], or starvation [105]. Although VBNC *Legionella* cells have low activity levels, they keep their virulence, and upon resuscitation within amoebae, they might become infectious for human cells [109]. For example, Schrammel et al. [105] demonstrated that a stable sub-population of VBNC *Legionella* was able to resist harsh environmental conditions for several months. Shaheen et al. [110] found that low temperatures triggered VNBC cell states, decreasing culturable counts of *L. pneumophila*. Yet the VBNC state is not a mechanism of replication, it is a critical asset for legionellae survival and adaptation to commonly used preventive and control practices. As such, VNBC *Legionella* cells represent an increased potential risk to human health that must be further studied and understood [111].

### 3.2. The Ecological Niches of Legionella—Protozoa and Biofilms

The relationship between protozoa (particularly amoebae) and legionellae is very diverse in nature [100], but in most cases, protozoa serve as an environmental habitat for *Legionella* replication [100,112]. Impressively, *Legionella* managed to resist amoebae digestion and succeeds in taking nutritional advantage from the host for its replication [22]. Similarly to what happens with human alveolar macrophages, when *Legionella* invades amoebae, it forms a unique protective compartment [2,113]. This vacuole does not follow the traditional endocytic pathway [22], and contrary to conventional phagosomes, they do not fuse with lysosomes or acidify [114]. While surrounded by the endoplasmic reticulum, the vacuole provides a nutrient-rich set of conditions that supports *Legionella* replication [114] to levels that increase legionellosis risk [115]. As nutrients are consumed and the depletion of the amino acid occurs, bacteria shift to a transmissive form, and where replication stops, bacteria become virulent [113] and are ready to escape to the bulk water and find a new host or favorable conditions for its replication. This refined *Legionella* life cycle that alternates between a replicative and a transmissive form encompasses several metabolic and physiological changes. This is probably one of the most relevant mechanisms that governs the growth and infectivity of bacteria in man-made systems [113].

The complexity of *Legionella* proliferation mechanisms in water systems becomes even more interesting when biofilms are considered [116]. Biofilms are microbial communities attached to surfaces and assembled in a matrix of self-secreted extracellular polymeric substances (EPS) [117]. Biofilms that form on real-field surfaces are most of the time not only composed of microorganisms and EPS, but encompass a miscellany of different biotic and abiotic material, including, for example, corrosion products, clay particles, or complex dissolved and colloidal matter [117,118,119]. The conceptual analogy that biofilms are the ‘city of microorganisms’ [120] illustrates the variety and sophistication of the relationships (such as cooperation or hostility) established by the microbial consortia in the biofilm. These microbial layers attached to surfaces have been, for several decades and for different reasons, one of the biggest concerns of water systems management [118,121,122].

Biofilms shelter a diverse community of microorganisms, including bacteria, fungi, algae, and protozoa [123]. Protozoa are important components or predators of the biofilms, affecting their structure and their internal complex feeding dynamics [124]. Murga et al. [125] demonstrated that *Legionella* spp. are able to persist in a laboratory biofilm of *Pseudomonas aeruginosa*, *Klebsiella pneumoniae,* and a *Flavobacterium* sp.; however, they are not able to replicate without the presence of *Hartmannella vermiformis*. The work by Declerck et al. [126] shows that the presence of the amoebae *Acanthamoeba castellanii* is important to spread *L. pneumophila* in a laboratory-simulated biofilm (from water distribution pipes) in a rotating annular reactor. Recently, Shahen et al. [110] proposed an interesting model for the association of *Legionella*–amoebae–biofilms. At first, biofilms and free-living amoebae growths are positively linked, and amoebae feed on (non-pathogen) bacteria in the biofilm. When the nutritional options become scarce and the ratio of amoebae to *Legionella* increases, amoebae enter a ‘must-feed-on-*L. pneumophila*’ mode, undergoing the formerly described growth/release-to-the-water cycles, liberating high concentrations of *L. pneumophila* in the bulk water. This model, in a broader sense, seems to corroborate the conclusions of van der Kooij et al. [127], who observed that *L. pneumophila* proliferation depends on host protozoan, and found out that pathogen growth was dependent on the biofilm concentration—reduced *Legionella* growth was also observed when biofilm concentration decreased. Additionally, the work by Kuiper et al. [128] shows that the intercellular growth of *L. pneumophila* in *Hartmannella vermiformis*, in a batch laboratory system, was the main proliferation mechanism in the biofilm. Very interestingly, the authors concluded that 90% of *H. vermiformis* was present in the biofilm and observed a positive relationship between the *Legionella* concentration in the system and the attached biomass amount, suggesting that controlling biofilm build-up can limit *L. pneumophila* proliferation.

On the other hand, studies with other biofilm models indicate that *L. pneumophila* might use the exogenous products (e.g., amino acids) of other environmental bacteria to support its replication [107,129]. Surman et al. [104] used a model water system to investigate whether *L. pneumophila* would replicate without a host protozoan. The authors’ conclusions suggest that intracellular replication is not mandatory for *Legionella* replication ‘as long as there are other bacterial species present’. This supports the findings of Taylor et al. [101], which highlighted the role and complexity of the different survival mechanisms that *Legionella* seems to be able to use, adapt, and persist in the water systems.

An exhaustive overview of the link between *Legionella* and biofilms or between *Legionella* and protozoa is out of the scope of the present review. The reader might find complementary important information about these topics in former works [30,31,100,101,114,124].

### 3.3. Bottlenecks of Real-Field Legionella Control

Whether *Legionella* can replicate in the biofilm without a host protozoan or not, it is consensual that biofilms are relevant sites for *Legionella* settlement in man-made water systems [27]. As a consequence of the biofilm life cycle or as a result of operational dynamics of the water system, part of the biofilms colonized with *Legionella* might be dislodged and, upon aerosolization, cause legionellosis events [23,27]. Furthermore, biofilm shelters its microbial community against external aggressions such as temperature changes or biocides [130]. For example, Giao et al. [131] used a two-stage chemostat to grow heterotrophic biofilms from drinking water and studied the effect of increasing chlorine dosages on *L. pneumophila* planktonic and sessile (biofilm) cells. The authors found that, regardless of chlorine presence (tested concentrations of 0.2 and 1.2 mgCl_2_/l), *L. pneumophila* could represent up to 25% of the total attached microbial community and that the total cell numbers of *Legionella* in the biofilm were not affected by the residue’s concentrations of biocide. These results agree with the conclusion of Wright et al. [132], who found that sessile populations were more resistant to the two tested biocides (Kathon and Bronopol) as compared with planktonic cells, emphasizing the extra protection conferred by biofilms [132]. Furthermore, the biofilm’s physical stability is highly relevant for the success of cleaning and disinfection procedures [133,134].

This puts great emphasis on proper biofilm management as part of an integrated approach to mitigate legionellosis incidence. Therefore, biofilm (often linked to dirtiness) control techniques are important components of legionellosis prevention [23,24,25]. However, at this point, a paradigmatic aspect typically arises: although effective water treatment programs against *Legionella* should focus on biofilms and planktonic bacteria [24], the indicative threshold action levels are only set for bacteria in the water (*Legionella* spp.) [23,24]. In practice, this might result in *Legionella* management procedures that are essentially grounded in occasional *Legionella* water sampling results, which follow an underlying logic of ‘non-detected’ vs. ‘detected’. Through this perspective, a ‘non-detected *Legionella* spp. result’ might be interpreted as ‘everything is OK’, while a positivity might indicate that something must be done or adjusted [10,135].

Grounding *Legionella* management on discrete planktonic heterotrophic bacteria counts and *Legionella* spp. screening is probably one of the main weaknesses of current preventive real-field practices. Counteracting and over-relying on such information biases the interpretation of the microbiological status of the system [10,27,28]. Firstly, water samples do not give representative information about the number of microorganisms in the system nor about the extent or location of the biofilm [136]. For example, Flemming et al. [123] estimated that 95% of the biomass present in drinking water distribution systems is attached to the walls rather than in the water. The under-representativeness of water samples is further illustrated in the works of Bonadonna et al. [137]. Bonadonna et al. [137] showed that the concentration of legionellae in biofilms from hot water networks was more than three orders of magnitude higher than the one recovered from the bulk water.

This point is further aggravated by discrete sampling, i.e., single snapshots in time of the microbiological status of the system [28]. For example, Bentham [27] found that in 25 of the 28 cooling towers sampled, there was no statistical relationship between *Legionella* culture results taken 2 weeks apart, demonstrating that the microbiological status of the system changes within a small timeframe (as compared to routine water sampling).

### 3.4. The Scientific Perpetuation of a Water Legionella-Sampling Approach

Not surprisingly, *Legionella* sampling in the water has been perpetuated in real-field practices, but also in scientific studies. Despite the limitations previously discussed, routine *Legionella* screening in the water provides an output that has a call-to-action significance (especially for culture methods) that is very relevant to assess the efficacy of proper *Legionella* water safety management [23,25,28].

Culture methods, such as the international standard ISO 11731 (*ISO 11731 ‘Water quality. Detection and enumeration of Legionella’*), have been standardized for several decades and are still considered the gold standard for *Legionella* screening in some reference documents [24]. Although they provide retrospective information (10 to 14 days to obtain a result) and underestimate the number of *Legionella* present in the water sample [138], the historical datasets and knowledge gained upon the use of culture methods over several decades (in distinct situations, including the investigation of legionellosis outbreak events) allowed the establishment of indicative thresholds of action according to the concentration of *Legionella* spp. in the water [25].

The advent of molecular techniques such as qPCR is providing an important boost to the study of *Legionella* ecology as they overcome some culture limitations [29,139]. These culture limitations are mostly linked to the following issues [29]: (i) *Legionella* cultivability is affected by the fastidious nature of the bacteria’s growth; (ii) the presence of other colonizing bacteria in the water sample may negatively affect the capacity of *Legionella* to grow in laboratory medium; (iii) *Legionella* VBNC cells [105] or *Legionella* inside vesicles (expelled from protozoa) are not detected; (iv) holding times between sampling collection and processing can lead to cultivability loss. On the other hand, qPCR detects DNA fragments that might belong to culturable, VBNC, and inactivated or even dead organisms, failing to distinguish between live and dead cells [29]. Due to the presence of inhibitory compounds, some water samples in CTs might also show qPCR inhibition, leading to false-negative results. Young et al. [29] estimated (based on five independent studies in CTs) that the inhibition fractions might be around 10%. Despite these limitations, the works of Young et al. [29] and Collins et al. [139] suggest that *Legionella* spp. qPCR is a good tool to use in routine monitoring, and they propose action and alert levels that can help to interpret GU (genomic units) of *Legionella* spp. per liter. More conservatively, Fisher et al. [140] advise the use of qPCR for rapid *Legionella* screening, where a PCR-negative result suggests no *Legionella* presence, and a positive output should require confirmation via culture method. Hopefully, the potentialities of molecular approaches will push the development of new methods for *Legionella* detection and quantification in situ and the design of simple-to-use and portable solutions for industrial application [141].

The lack of standard practices for biofilm sampling and analysis [32,33], even for research purposes, also contributes to this water screening perpetuation. Swabbing the surface is often used with the aim of analyzing *Legionella* at the biofilms [70,76], yet the scope of the standard application does not include biofilm sampling. Swab sampling is usually based on the international standard ISO 18593-2004 (*ISO 18593:2004 ‘Microbiology of food and animal feeding stuffs—Horizontal methods for sampling techniques from surfaces using contact plates and swabs’*). However, swab sampling aims to assess the microbial load on surfaces (mostly for food safety purposes) rather than sample or examine the biofilm in industrial water systems. Swab sampling destroys the biofilm structure, and measuring the swabbed area is often an unfeasible task [25]. However, in the absence of a more suitable approach, it is recommended for surface screening purposes related to *Legionella* [23].

*Legionella*’s specific environmental monitoring is still very limited and does not reflect the complex interactions within biofilms and protozoa. Why, however, does this still happen? Why is research so reluctant to bridge this gap and start including protozoa and biofilms in standard *Legionella* works? Do we have the tools and methods, but are they still not fully explored/understood? Or do we have to find new solutions for old problems? This dilemma is very well illustrated when the added value of online biofilm monitoring tools is compared with their effective use.

### 3.5. Online Biofilm Monitoring—An Unmet Need or an Unexplored Solution?

Online, continuous, non-destructive biofilm (and other deposits) monitoring appears as an important tool to assess, and prevent in a timely manner, build-up/detachment events, as well as to evaluate the efficacy of the applied countermeasures [142].

The works of Janknecht and Melo [143], Flemming [34], and Nivens et al. [144] provide interesting insights into biofilm monitoring approaches, discussing available techniques, their physical principles, and their advantages and disadvantages. Among the extended list of technologies reported in the literature, several are suitable for online monitoring in industrial systems [143]. Furthermore, some of these state-of-the-art technologies have been successfully tested and are commercially available for implementation in real-field water systems [145]. Despite the potentialities associated to each biofilm monitoring technique and their contribution to improved early-warning biofouling management, the water treatment industry/sector does not seem to have a clear strategy for their adoption (authors’ personal experiences). This happens because interpreting the sensor’s output information is often complex, requires specialized know-how [34], and becomes a serious barrier for their integration into the water system process. If integration in real-field systems fails, the monitoring potential for the water management program vanishes and it becomes just another setting that a system’s manager must supervise. This agrees with Flemming’s [34] arguments that the industry is still not committed to the optimization and validation of such early-warning tools, which, as explained, require a long timeframe and interdisciplinary work for their validation. At the end of the day, legislation might impose the adoption of online biofilm sensors but, to do so, science must strengthen the arguments about the potentials of complementary surface monitoring, not only for biofilm management but also for legionellosis prevention. Thus, following for example the conclusions of Kuiper et al. [128], if the biofilm is under continuous supervision and control, legionellosis prevention increases.

Reflecting on the questions previously enunciated, we might conclude that the tools are there and they have intrinsic potential, but academia and industry are not able to coordinately collaborate and fully demonstrate their added value. Following this rationale, the next section will discuss some ideas on how to build an integrated approach that allows a complementary study of *Legionella* ecology in real-field systems, which can be optimized and used in the future to enhance prevention in engineered water systems.

## 4. New Pathways to Build an Integrated and Effective *Legionella* Surveillance Strategy in Water Systems

Effective *Legionella* management needs to be an integrated process [23], adaptable to changes and grounded in consistent information about the water treatment critical issues. This process is conceived as a direct call to ‘keep an eye at the whole picture’, rather than just to ‘be focused on isolated pieces of the puzzle’. To meet the ambitious goal of building more integrated *Legionella* prevention practices, a paradigm shift is needed. As previously discussed, the intricate level of interactions among *Legionella* and the vast community of microorganisms in the bulk water and in the biofilm is scientifically very challenging and requires a ‘greater focus on total system ecology rather than on individual bacterial-protozoan interactions’ [101]. Some other authors [8,20] emphasize that improvements in legionellosis mitigation practices at engineered systems are very dependent on a broader understanding of legionellae ecology.

### 4.1. An Integrated Monitoring Physical Model for Legionella Study and Control in Real Systems

**Figure 1:**
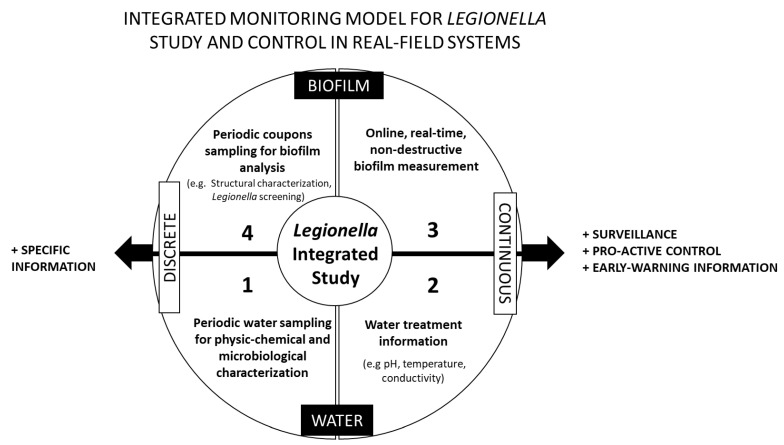
Integrated monitoring conceptual model for *Legionella* study and control in field-based systems. The model proposes four complementary sets of information: water (1 and 2) and biofilm (3 and 4) monitoring, discretely sampled (1 and 4) and continuously measured (2 and 3). Continuous information will enhance pro-active control and surveillance, based on early-warning information, while discrete information will allow to gain more specific information about *Legionella* ecology.

One feasible approach to gain this knowledge, while tracking operational features of the systems, is the combination of complementary monitoring methods, which include (a) online, continuous information and discrete sensing; (b) surface and water monitoring; (c) biofilm and *Legionella* analysis. Even though the development of such an idea can follow different pathways and certainly requires wider scientific reflection/discussion, we propose, for illustration purposes, an integrated monitoring model for *Legionella* study at field-based systems (Figure 1). This model aims to catalyze a joint discussion on a renewed *Legionella* management strategy, which can be optimized under the scope of field studies for later adoption at water utilities. Here, we will only focus on the macro perspective of the model rather than on overviewing specific methodologies, since those will depend on several items, including the sort of water system under study.

The conceptual model proposed in Figure 1 relies on the idea that *Legionella* control will be as effective as we manage to gain a broader perspective on the overall ecology of *Legionella*. Surveillance and pro-active control driven with online, continuous measurements are essential for effective *Legionella* mitigation practices, and specific information is key for enhancing understanding about *Legionella* overall ecology. Under these assumptions, four complementary sets of information were foreseen.

*1st Set of Information: Water—Discrete Sampling*

The first set of information is related to the routine monitoring approach, focused on periodic water sampling for physical, chemical, and microbiological characterization. This also includes *Legionella* spp. and *L. pneumophila* detection and quantification. Recently, Walker et al. [146] reviewed current *Legionella* testing methods, and LeChevallier [147] proposed an interesting guidancefor the development of a *L. pneumophila* monitoring plan for water utilities. Both works are of great importance to the implementation of improved routine *Legionella* monitoring procedures. Furthermore, given the role of protozoa in the overall *Legionella* ecology and virulence [113], it seems to be very important to include their analysis under this first level of monitoring. This also embraces with the findings of Shaheen et al. [148], who suggest that monitoring free-living amoebae can be useful to predict the ‘possible imminent high occurrence of *Legionella*’ in engineered water systems. Protozoa are not detected through traditional bacteriological methods, and the detection of a large diversity of free-living protozoa can be a challenging and laborious task [124]. This is demonstrated, for example, in the work of Valster et al. [149], who found that different protozoan communities developed in duplicated samples (samples from different water settings). Nisar et al. [35] discuss the relevance of molecular techniques such as PCR and fluorescence in situ hybridization (FISH) for *Legionella* and protozoan screening in environmental water samples. In this work, the authors also came across the conclusion that, in potable water systems (including hospitals), *Vermamoeba* and *Acanthamoeba* were the hosts predominantly associated with *L. pneumophila.* This also raises the possibility of selecting some specific protozoa indicators that might be linked to *L. pneumophila*. For example, the review conducted by Lau et al. [30] might be a great starting point for this discussion, since it systematizes the protozoa species (mostly amoebae) found to host *Legionella* species in drinking water settings.

*2nd Set of Information: Water—Continuous Monitoring*

The second set of information is related to standard water treatment parameters that will directly or indirectly reflect the performance of the control measures [150], including, for example, pH, conductivity, temperature, flow, critical pumps operation, and biocidal residue (if applicable). This also aligns with the WHO (World Health Organization) guidelines [23], which state that ‘operationally, control measures, (…) should be monitored online’. The need to reinforce operational monitoring is also stressed in the recently revised European Directive (2020/2181) on the quality of water for human consumption [26]. An online, real-time dataset of these parameters enables the timely identification and correction of punctual deviations to the established operational limits [23,26,54], avoiding situations that can favor *Legionella* proliferation. For example, Whiley et al. [151] reported real-time monitoring of the temperature and flow in the thermostatic mixing valves of water distribution networks as an interesting surveillance strategy to detect changes in water quality, as well as to identify hazardous situations regarding different opportunistic pathogens, including *Legionella*. This continuous information would be an important complement to well-established water routine sampling, as discussed in previous sections since it raises the opportunity to keep continuously an eye on the system in between samplings and while microbiological analysis is being processed. This information would also serve for registration purposes (an essential asset of a proper *Legionella* prevention plan) [24].

*3rd Set of Information: Biofilm—Online Monitoring*

As formerly discussed, the potential of online, continuous, non-destructive biofilm monitoring can be determinant to establish a proactive, informated-based water management [34]. Flemming [34] systematized the features of an ideal online, real-time biofouling monitoring sensor able to provide information about the biofilm: location and extent, quantity (mass, thickness), nature of the deposit (organic/inorganic, biological/non-biological, chemical composition), the kinetics of deposit formation, and removal. Additionally, such monitoring tools should be applied to a large monitoring area and should be low cost and easy to handle. Due to this long and very specific list of features, it is very unlikely that a unique sensor meets all these requirements at once. As such, combining different monitoring tools into an ‘all-in-one’ solution is probably the most feasible way to strengthen the arguments for their routine implementation. This ‘all-in-one’ setup should combine a selection of tools that are suitable for real-field operation and that provide distinct (but complementary) output information about biofilm deposits.

Regarding *Legionella* prevention, it seems plausible to accept that both the biofouling extent and nature (biotic/abiotic) of the attached layers are important parameters to assess. Measuring biofilm build-up/removal kinetics can provide important insights on ‘how fast is the biofilm being formed/removed’ and ‘how far will the stabilization plateau be achieved’. This concept is somewhat similar to the ‘Biofouling Formation Potential’ described by van der Kooji et al. [127], yet applied to a different measuring unit. Those two indicators (kinetics and maximum biofilm amount) will provide information about the biofilm formation potential of the system and the biofilm extent, respectively. Both the ‘stabilization plateau’ and ‘threshold of interference’ [152], as well as biofilm kinetics, depend on the particular water system and its specific operating conditions [34]. As such, for a given system, at a given representative location, an increased build-up rate or an unexpected sloughing-off event (which can bring *Legionella* back into the bulk water) are certainly examples of early-warning calls that something in the standard operation has changed (even though that can be a planned change). Similarly, removal rates can be used to assess the efficacy of implemented countermeasures. For example, Pereira et al. [153] reported the use of a surface sensor technology [154] to monitor in real-time the formation/removal of biofouling layers, identifying proactively processual changes in the bypass of a cooling water system.

Evaluating the nature (biotic/abiotic) of the biofilm layer can be important for assessing and adjusting the efficacy of microbial control programs [142], with the aim of keeping microbial growth at the surface under control. For example, the commercially available Alvim sensor [155]—an online, electrochemical sensor—was successfully used in industrial water settings to follow the biofilm growth and to optimize cleaning procedures. Monitoring the nature of the deposit will be particularly relevant in finding out how biotic and/or abiotic attached layers affect *Legionella* persistence. Another promising tool is the OnGuard^TM^ analyzer, which has been successfully used to optimize the biocidal program of a cooling water system, based on the detection of biofouling formation/removal kinetics [156]. This analyzer can also provide information about the nature of the attached deposit [156].

*4th Set of Information: Biofilm—Discrete Sampling*

To gain detailed information that can enhance *Legionella* ecology understanding, surface online monitoring must be complemented with biofilm discrete sampling, followed by a detailed analysis and characterization, including *Legionella* screening. For that, the inclusion of biofilm sampling probes (or coupons), which can be periodically removed over time, might be a suitable approach. Some overviews on biofilm formation devices suitable for industrial application can be found in the works of [157] or [158], for example. Some interesting solutions for biofilm formation studies are the Flow Cell system [154,159,160] or the Modified Robbins Devices [161], which are very well characterized in the laboratory in terms of operation and hydrodynamics and have been successfully used in the study of biofilms in full-scale water systems.

The work of Azeredo et al. [32] is a good starting point to choose which analytical techniques for biofilm characterization best fits a study’s purposes. Apart from the standard methods focused on biofilm physiology and the composition of the attached layers, we emphasize the role that structural characterization plays in the control of *Legionella*. Several arguments support this suggestion: (a) protozoa have a significant impact on ‘shaping’ biofilm architectures [124], (b) biofilm structure affects the efficacy of countermeasures [162], (c) sloughing-off events are more likely to occur when heterogeneity increases [163]. As such, evaluating structural changes in real-field systems can inform on biofilm and protozoa interactions, with a visible influence on *Legionella* control.

### 4.2. Representativeness—Worst Case Scenario Conditions

A critical issue in the implementation of the conceptual model proposed herein is representativeness since most of the key points regarding biofilm build-up and *Legionella* settlement are not accessible for sensor installation or sample collection. Engineering a bypass monitoring platform, combining the different monitoring sets of information, and operating under worst-case scenario conditions, can overcome this representativeness limitation. Worst-case conditions are accepted as part of *Legionella* monitoring plans, in case it is impossible to overcome physical or processual limitations [24]. For example, it is recommended that routine water sampling might be collected at the time (for example, before biocide dosage) and place (warmer temperatures) that represent the highest risk for *Legionella* settlement in the system [24]. The idea of a bypass monitoring platform relies on the assumption that if the water treatment favors (or not) biofilm formation/removal and *Legionella* settlement, it will preferentially occur and be detected at the monitoring platform. As such, properly testing the worst-case conditions becomes a crucial step. Since both biofilms and *Legionella* are affected by, for example, hydrodynamics, temperatures, and surface materials [23,31], these parameters can be carefully chosen and set at the bypass monitoring platform to mimic the critical spots of the main system.

The complexity of this conceptual monitoring model demands a wise balance between a ‘perfect monitoring solution’ and a fit-to-purpose, real-field implementableone. The definition of consistent data flows (of process and biofilm indicators), and the ability to transform such data into meaningful information, can be a decisive step towards a successful approach. This would meet the expectation drawn by Fields et al. [22], for example, that ‘Computer-based reporting systems may one day provide a means of conducting timely surveillance’. A final real-field implementable solution will have to bridge the gap between the approach (what should be done) and implementation (what can actually be done).

### 4.3. Final Disclaimer

While the ideas discussed in this final section might sound very exploratory, they aim to bring together existing tools and new elements to the discussion and studies around *Legionella* management in man-made water systems. The conceptual monitoring model proposed in Figure 1 aims to encourage the strengthening of *Legionella* monitoring procedures by integrating different approaches that can provide a broad perspective on *Legionella* ecology and improve its surveillance in water systems. This model is especially important in the framework of real-field studies discussed in Section 2, which are a great opportunity to bridge knowledge across disciplines while reinforcing scientific outputs towards new standardized and integrated methodologies. Integrated data monitoring and analysis, which can provide early-warning information, will certainly build more resilient real-field *Legionella* control practices and strengthen field-based scientific outputs.

## 5. Conclusions

*Legionella* control at water systems is a multivariable problem. It is unfeasible to assume that *Legionella* might be eradicated from water systems; therefore, prevention assumes great relevancy. Field-based trials are an important component of *Legionella* study. However, these studies are traditionally focused on assessing *Legionella* ecology in the bulk water, often disregarding the role of protozoa and biofilms as critical ecological niches for *Legionella* growth, infectivity, and perseverance in water systems. Improved, consistent, and adaptable-to-change *Legionella* management procedures require a great focus on the total ecology of the system and a wider convergence between engineering tools and microbiological approaches. To boost this discussion, an integrated monitoring model for *Legionella* study and control at field-based systems is proposed here. This model is grounded in the combination of four complementary sets of information and is expected to bridge the gap between scientific approaches and real-field needs, so as to enhance *Legionella* understanding and pro-active surveillance in the water systems.

## Data Availability

Not applicable.
